# *Lycium barbarum* Polysaccharides Alleviate Ethanol‐Induced Liver Injury by Activating PPAR‐α and Inhibiting NLRP‐3/Caspase‐1‐Mediated Pyroptosis

**DOI:** 10.1002/fsn3.70172

**Published:** 2025-04-18

**Authors:** Le‐bin Cai, Quan Zhou, Na Tao, Wen‐zhong Chen

**Affiliations:** ^1^ Department of Infectious Disease, Guangzhou First People's Hospital, School of Medicine South China University of Technology Guangzhou Guangdong China; ^2^ Department of Nursing, Guangzhou First People's Hospital, School of Medicine South China University of Technology Guangzhou Guangdong China; ^3^ Department of Cardiovascular Medicine, Guangzhou First People's Hospital, School of Medicine South China University of Technology Guangzhou Guangdong China

**Keywords:** alcoholic liver disease, hepatoprotection, *Lycium barbarum* polysaccharides, NLRP‐3 signaling, PPAR‐α, pyroptosis

## Abstract

This Research Aimed to Discuss the Protective Mechanism of *Lycium barbarum* Polysaccharides (LBPs) Against Ethanol (EtOH)‐caused Hepatocellular Damage. Normal human hepatocytes (L‐02 cells) were processed with 100 μg/mL EtOH to simulate liver injury, followed by treatment with LBPs at different concentrations (12, 24, 48 μg/mL) to determine the optimal dose. Cells were divided into the control, EtOH, EtOH+LBP‐treated, and EtOH+LBP‐treated with siRNA against PPAR‐α groups. To evaluate treatment effects, the MTT assay was utilized for measuring cell viability, succeeded by the assessment of liver injury markers (ALT, AST, TG) and inflammatory cytokines (IL‐1β, TNF‐α, and IL‐6). Besides, the GSDMD, NLRP‐3, caspase‐1, and PPAR‐α protein levels were analyzed via western blotting. Relative to the Control group, EtOH exposure remarkably decreased cell viability, increased TG, AST, and ALT levels (*p* < 0.01), and induced cell damage and lipid accumulation. It also elevated inflammatory cytokine levels and triggered pyroptosis (*p* < 0.01). However, LBP treatment alleviated EtOH‐induced damage, reduced lipid accumulation, inhibited pyroptosis‐related protein expression, suppressed inflammatory responses, and upregulated PPAR‐α protein expression (*p* < 0.01). LBPs can alleviate EtOH‐induced L‐02 cell injury, lipid accumulation, inflammatory response, and pyroptosis. The mechanism is possibly associated with inhibiting NLRP‐3/caspase‐1‐mediated cell pyroptosis by activating PPAR‐α expression, thus protecting hepatocytes from injury.

## Graphical abstract

Our study suggested that LBPs protected against EtOH‐induced liver injury through multiple mechanisms, including the inhibition of inflammatory responses, suppression of NLRP‐3/caspase‐1‐mediated pyroptosis, and activation of the PPAR‐α signaling pathway. These findings contribute to the understanding of the therapeutic potential of LBPs in managing ALD and provide a foundation for future research into their clinical applications.

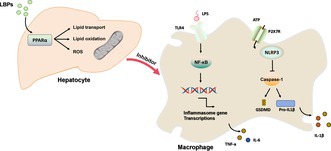

**Abbreviations**
ALD: Alcoholic liver disease
ALT: Alanine transaminase
AMPK: AMP‐activated protein kinase
AST: Aspartate aminotransferase
ELISA: Enzyme‐linked immunosorbent assay
EtOH: Ethanol
GSDMD: Gasdermin D
IL: Interleukin
LBPs:
*Lycium barbarum* polysaccharides
MTT: 3‐ (4,5‐dimethylthiazol‐2‐yl)‐2,5‐diphenyltetrazolium bromide
NASH: Non‐alcoholic steatohepatitis
NLRP‐3: Nod‐like receptor protein 3
PPAR‐α: Peroxisome proliferator‐activated receptor‐alpha
qRT‐PCR: Quantitative reverse transcription polymerase chain reaction
TG: Triglycerides
TG: Triglycerides
TNF‐α: Tumor necrosis factor alpha

## 1 Introduction

As a pivotal metabolic organ, the liver is critical in detoxifying xenobiotics, but this function also makes it highly vulnerable to damage from various toxins (Lotto et al. 2023; Roohani and Tacke 2021). Liver injury can result from exposure to toxic drugs (such as isoniazid, rifampicin, and acetaminophen), heavy metals (such as cadmium, chromium, and arsenic), and environmental chemicals like paraquat (Seyoum 2023). Among these factors, excessive alcohol intake is a major contributor to liver disease globally, particularly in China, where rapid economic development and lifestyle changes have led to increased alcohol consumption. Excessive ethanol (EtOH) intake may cause alcoholic liver disease (ALD), a progressive condition that can lead to liver failure and death if untreated (Malnick et al. 2022; Wu et al. 2023). Despite advances in ALD research, current treatment strategies remain limited, primarily relying on lifestyle interventions and supportive therapies, with no widely effective pharmacological treatments available (Khan et al. 2025). Thus, identifying novel therapeutic agents and elucidating their mechanisms is essential for developing effective ALD interventions.

Recent studies have shown that natural polysaccharide‐based formulations could enhance immune responses and modulate inflammatory pathways (Abbas et al. 2024; Shahzad et al. 2024) In this context, *Lycium barbarum* polysaccharide (LBP) has been highly regarded in Traditional Chinese Medicine for its health benefits, including its antioxidative, hepatoprotective, and anti‐inflammatory properties. LBP can offer therapeutic benefits in various liver injury models (Tian et al. 2019), such as carbon tetrachloride‐induced liver injury (Chiang and Chao 2018), cadmium‐induced liver injury (Varoni et al. 2017), non‐alcoholic steatohepatitis (Xiao et al. 2018), and liver injury triggered by heavy metal (Yan et al. 2019). These studies indicate that LBP may exert protective effects by modulating pathways associated with apoptosis, oxidative stress, and mitochondrial dysfunction (Varoni et al. 2017; Yan et al. 2019). For instance, using the AMP‐activated protein kinase signaling, combining LBPs with aerobic exercise activates peroxisome proliferator‐activated receptor‐alpha (PPAR‐α), which reduces lipid accumulation and inflammation in non‐alcoholic steatohepatitis models (Li et al. 2022).

However, while previous studies have demonstrated the hepatoprotective effects of LBPs, the specific molecular mechanisms underlying their action in ALD remain unclear. Studies have reported that LBPs reduce oxidative stress and lipid accumulation, but their influence on inflammatory cell death pathways such as pyroptosis has not been systematically examined (Li et al. 2024). Moreover, LBP's precise role in modulating key ALD‐related signaling pathways, including PPAR‐α and inflammasome activation, remains largely unexplored. Given these uncertainties, it is crucial to investigate how LBPs influence pyroptosis and inflammation in ethanol‐induced liver injury.

As an inflammatory type of programmed cell death, pyroptosis has recently been highlighted as a central mechanism in ALD (J. Wu et al. 2019). Mediated by inflammasomes such as nod‐like receptor protein 3 (NLRP‐3), pyroptosis leads to inflammatory cytokine release, including IL‐1β, and caspase‐1 activation, exacerbating liver injury and inflammation (Gao et al. 2018). Notably, PPAR‐α activation has been shown to inhibit NLRP‐3‐mediated pyroptosis, thereby reducing liver inflammation (Jiao et al. 2023; Wang et al. 2021; Zhang et al. 2023). Given that LBPs have been previously linked to PPAR‐α activation and anti‐inflammatory effects, it is reasonable to hypothesize that they may exert hepatoprotective effects through PPAR‐α‐mediated suppression of NLRP‐3/caspase‐1 pyroptosis. However, this mechanistic relationship has not yet been confirmed in ethanol‐induced liver injury models.

This study aims to fill this research gap by investigating the hepatoprotective effects of LBP against ethanol‐induced liver injury through modulation of the PPAR‐α/NLRP‐3/caspase‐1 pathway in a human L‐02 cell model. Specifically, it examines whether LBP can activate PPAR‐α and inhibit pyroptosis, thereby alleviating liver inflammation and injury. Understanding these mechanisms could offer a foundation for applying LBP clinically to treat ALD and other inflammatory liver diseases.

## 2 Materials and Methods

### 2.1 Cell Culture

The National Collection of Authenticated Cell Cultures (No. Cs‐371) provided L‐02 cells, which were subsequently validated through Short Tandem Repeat analysis. Cultivation of the cells was conducted in an incubator set to 37°C with 95% humidity and 5% CO_2_. The culture medium used was Dulbecco's Modified Eagle Medium‐high glucose (Hyclone, USA) with fetal bovine serum (10%, Hyclone, USA) as well as penicillin/streptomycin (1%, Hyclone, USA). Cells were passaged when 70%–80% confluent. Routine mycoplasma testing was performed to ensure cell line integrity.

### 2.2 Cell Treatment and Transfection

L‐02 cells in the exponential phase were harvested and assigned to five groups: (1) EtOH group: Cells underwent 24‐h exposure to 100 μg/mL EtOH for 24 h; (2) Control group: cells underwent conventional culture without any treatment; (3) LBP group: After a 24‐h exposure to EtOH (100 μg/mL), cells received additional 24‐h treatment with 48 μg/mL LBP; (4) LBP + si‐NC group: Negative control siRNA (si‐NC) was transfected into cells for 24 h, followed by 24‐h exposure to EtOH (100 μg/mL) and treatment with LBP (48 μg/mL) for the next 24 h; (5) LBP + si‐PPAR‐α group: Cells underwent PPAR‐α siRNA transfection for 24 h, followed by a 24‐h treatment with LBP (48 μg/mL). LBP and EtOH employed in this study were supplied by Natural Field Bio‐Technique Co. Ltd. (Shaanxi, China) and Sigma‐Aldrich, respectively.

For each experimental group, the corresponding plasmids—si‐NC (5′‐UUCUCCGAACGUGUCACGUTT‐3′) and si‐PPAR‐α (5′‐UGAACUUCAUGGCAAAAUCAA‐3′)—were purchased from Shanghai Genechem Co. Ltd. The plasmids were diluted in the culture medium and transfected into hepatocytes using an electroporation technique optimized for liver cells. Specifically, cells in the logarithmic phase were mixed with the respective plasmid solutions (si‐NC and si‐PPAR‐α, from Shanghai Genechem Co. Ltd.) and transferred to electroporation cuvettes. Electroporation was carried out with a single pulse of 250 V for 20 milliseconds using a Bio‐Rad electroporator. Following electroporation, cells were transferred immediately to pre‐warmed media and allowed to recover for 12 h before subsequent treatment with EtOH or LBP as outlined in the study protocol. After the cells adhered to the cup wall, all groups except for the control group were treated with 100 mmol/L EtOH (Guangzhou Shunfeng Technology Co. Ltd.) (Neuman et al. 2017). Then, L‐02 cells in each group were mediated via LBPs at varying concentrations (48, 24, and 12 μg/mL) (prepared in this experiment) (Xiao et al. 2013; Xu et al. 2017).

### 2.3 3‐(4,5‐Dimethylthiazol‐2‐Yl)‐2,5‐Diphenyltetrazolium Bromide (MTT) Assay

A 96‐well plate was selected for the seeding of L‐02 cells (5 × 10^3^ cells/well and 100 μL/wells), followed by a 24‐h culture period. After 24 h, each well received 10 μL of MTT solution (0.5 mg/mL, Abcam), followed by a 4‐h incubation. After aspirating the culture medium, formazan crystals were dissolved by adding 150 μL of dimethyl sulfoxide. Ultimately, a microplate reader was applied to determine the absorbance at 490 nm.

### 2.4 Measurement of TG, AST, and ALT

A 24‐well plate was selected for the inoculation of L‐02 cells (5 × 10^4^ cells/mL and 500 μL/well) and cultured for 24 h before treatments. Supernatants were harvested following a 4‐h incubation and analyzed for alanine transaminase (ALT), aspartate aminotransferase (AST), and triglycerides (TG) levels using an automatic biochemical analyzer (Shanghai Kehua Bioengineering Co. Ltd.). Nanjing Jiancheng Bioengineering Institute provided the detection kits for TG (Cat#A0008085), AST (Cat#A0008084), and ALT (Cat#A0008086).

### 2.5 Elisa

L‐02 cells were treated as described above, then harvested and lysed after 24 h. Lysates underwent 15‐min centrifugation at 4°C, followed by a collection of the supernatant for analysis. As instructed by the manufacturer, ELISA kits (Nanjing Jiancheng Bioengineering Institute) were employed to detect the interleukin (IL)‐1β (Cat#A0008092), tumor necrosis factor‐alpha (TNF‐α, Cat#A0008097), and IL‐6 (Cat#A0008095) levels.

### 2.6 Western Blotting

Seeding of L‐02 cells in a 6‐well plate was followed by a 24‐h culture period. After treatment, cell lysis was performed with RIPA lysate, and a bicinchoninic acid kit (Beyotime) was adopted for measuring protein concentrations. Sodium dodecyl sulfate‐polyacrylamide gel electrophoresis was employed for separating proteins, followed by their transfer to nitrocellulose membranes. Primary antibodies were employed for overnight incubation at 4°C after blocking the membranes with 5% skim milk for 1 h at ambient temperature. The primary antibodies included NLRP‐3 (Cat#ab263899, 1: 1000), caspase‐1 (Cat#ab207802, 1: 1000), GSDMD (Cat#ab219800, 1: 2000), cleaved caspase‐1 (Cat#ab207804, 1: 1000), GSDMD‐N (Cat#ab215203, 1: 1000), PPAR‐α (Cat#ab126285, 1: 1000), and GAPDH (Cat#2118S, 1: 1000). TBST washing was followed by incubation of membranes for 1 h at ambient temperature using Goat anti‐Mouse IgG (H + L) Secondary Antibody (Thermo Fisher, Cat#PA1‐28555, 1: 10000) or Goat anti‐Rabbit IgG (H + L) Secondary Antibody (Thermo Fisher, Cat#31460, 1: 10000). The membrane underwent three washes with TBST, after which protein bands were visualized using ECL substrate and analyzed with ImageJ software.

### 2.7 qRT‐PCR

TRIzol reagent (Invitrogen, Carlsbad, CA, USA) was utilized for extracting RNA, and the High‐Capacity cDNA Reverse Transcription Kit (Applied Biosystems, Foster City, CA, USA) was adopted for reverse transcription into cDNA, as per the manufacturer's protocol. Using SYBR Green PCR Master Mix (Applied Biosystems, Foster City, CA, USA), qRT‐PCR was conducted on a StepOnePlus Real‐Time PCR System (Applied Biosystems, Foster City, CA, USA). Thermal cycling conditions included an initial denaturation at 95°C for 10 min, followed by 40 cycles of 15 s at 95°C for denaturation and 1 min at 60°C for annealing/extension. To quantify and normalize gene expression, the 2^−ΔΔCT^ method was utilized, with GAPDH as the internal control. Listed below are the primer sequences: PPAR‐α: 5′‐GCACTTGTGAAAACGGCAGT‐3′ (reverse), 5′‐TCGGCGAACTATTCGGCTG‐3′ (forward); GAPDH: 5′‐GCGGCACGTCAGATCCA‐3′ (reverse), 5*'*‐CATGGCCTTCCGTGTTCCTA‐3′ (forward).

### 2.8 Statistical Methods

For statistical analysis, SPSS 24.0 software was adopted. The Shapiro–Wilk test was employed for evaluating data normality. For data with normal distribution, a one‐way analysis of variance followed by Tukey's post hoc test with Bonferroni correction was applied for multiple comparisons. The Kruskal–Wallis test was utilized for analyzing non‐normally distributed data. Mean ± standard deviation was applied for presenting normally distributed data, and median and interquartile range for non‐normally distributed data. *p* < 0.05 represented the statistical significance threshold.

## 3 Results

### 3.1 LBPs Alleviate EtOH‐Induced Lipid Accumulation and Damage in L‐02 Cells

**FIGURE 1:**
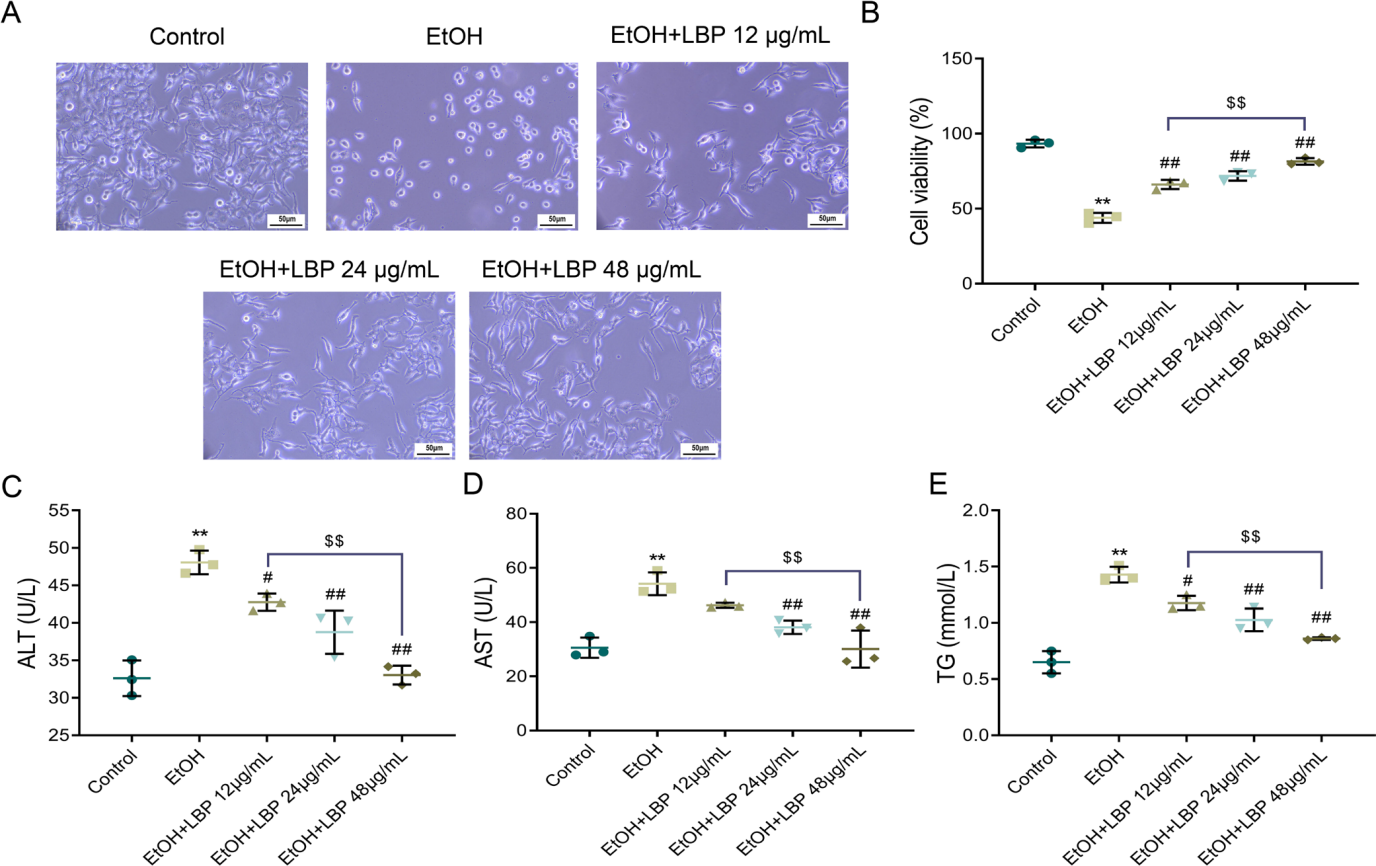
LBPs alleviate ethanol‐induced damage and lipid accumulation in L‐02 cells. (A) Representative micrographs of L‐02 cells depicting morphological changes in each group. Scale bar = 50 μm. (B) MTT assay for detecting the cell viability in each group. (C–E) Biochemical assays measuring levels of ALT (C), AST (D), and TG (E) in cells of each group. Data representation: Mean ± SD (*n* = 3). One‐way ANOVA followed by Tukey's post hoc test with Bonferroni correction was used for multiple comparisons. **p < 0.01 versus Control group; ^#^ *p* < 0.05, ^##^ *p* < 0.01 versus EtOH group; ^$$^ *p* < 0.01 versus EtOH+LBP 12 μg/mL group. ALT, alanine transaminase; AST, aspartate aminotransferase; EtOH, ethanol; LBP, *Lycium barbarum* polysaccharide; TG, triglycerides.

An initial evaluation was conducted on the potential cytotoxicity of LBPs in L‐02 cells and their ability to alleviate EtOH‐induced damage and lipid accumulation to investigate LBPs' protective effects against liver injury triggered by EtOH. Referring to a previous study (Wang et al. 2020), LBPs did not cause cytotoxicity at concentrations of 48, 24, and 12 μg/mL in the absence of EtOH. Morphological examination revealed distinct changes under various treatment conditions. The control group displayed typical hepatocyte morphology, indicative of healthy cell status. In stark contrast, EtOH‐treated cells presented with signs of distress, including cellular shrinkage and expanded intercellular spaces, which are markers of EtOH‐induced damage. Morphological alterations were progressively reversed in response to LBP treatments at increasing concentrations (12, 24, and 48 μg/mL). Notably, cells treated with 48 μg/mL LBP exhibited morphology closely resembling that of the control group, suggesting an obvious protective effect against EtOH‐mediated damage (Figure 1A).

Consistent with morphological findings, the EtOH group exhibited a substantial decrease in cell viability and an increase in ALT, AST, and TG levels as opposed to the control group. LBP treatments significantly enhanced cell viability and reduced ALT, TG, and AST levels concentration‐dependently (*p* < 0.01, Figure 1B–E). Notably, the higher cell viability and more pronounced reductions in ALT, AST, and TG levels were observed with the 48 μg/mL LBP compared to the 12 μg/mL concentration (*p* < 0.01), indicating its superior efficacy in mitigating EtOH‐induced hepatocellular injury and lipid accumulation. The aforementioned outcomes suggested that LBPs significantly protected L‐02 cells from EtOH‐induced damage and lipid accumulation in L‐02 cells.

### 3.2 LBPs Inhibit EtOH‐Induced Inflammation in L‐02 Cells

**FIGURE 2:**
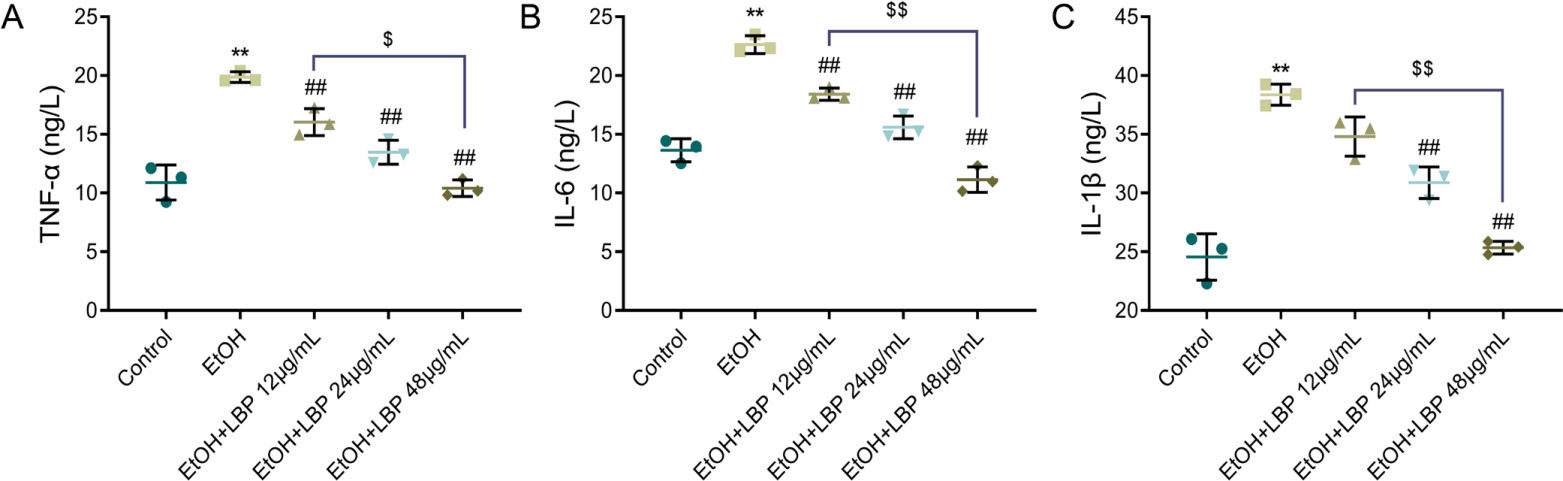
LBPs suppress ethanol‐caused inflammation in L‐02 cells. (A–C) *ELISA* results showing levels of TNF‐α (A), IL‐6 (B), and IL‐1β (C) in cells of each group. Data representation: Mean ± SD (*n* = 3). One‐way ANOVA followed by Tukey's post hoc test with Bonferroni correction was used for multiple comparisons. **p < 0.01 versus Control group; ^##^ *p* < 0.01 versus EtOH group; ^$^ *p* < 0.01, ^$$^ *p* < 0.01 versus EtOH+LBP 12 μg/mL group. EtOH, ethanol; IL, interleukin; LBP, *Lycium barbarum* polysaccharide; TNF‐α, tumor necrosis factor alpha.

Following the assessment of LBPs' protective effects on cell morphology and viability, we further investigated whether LBPs could mitigate EtOH‐induced inflammation in L‐02 cells. The EtOH group displayed markedly raised levels of pro‐inflammatory cytokines IL‐1β and TNF‐α as opposed to the control group. However, relative to the EtOH group, a notable, concentration‐dependent decline in these cytokine levels was observed following LBP treatment (*p* < 0.01, Figure 2). More importantly, 48 μg/mL LBP exhibited superior anti‐inflammatory effects compared to the lower dose (*p* < 0.05 or *p* < 0.01). Therefore, LBP was effective in reducing EtOH‐induced inflammation in L‐02 cells, highlighting its potential anti‐inflammatory properties.

### 3.3 LBPs Inhibit EtOH‐Induced Expression of Pyroptosis‐Related Proteins in L‐02 Cells

**FIGURE 3:**
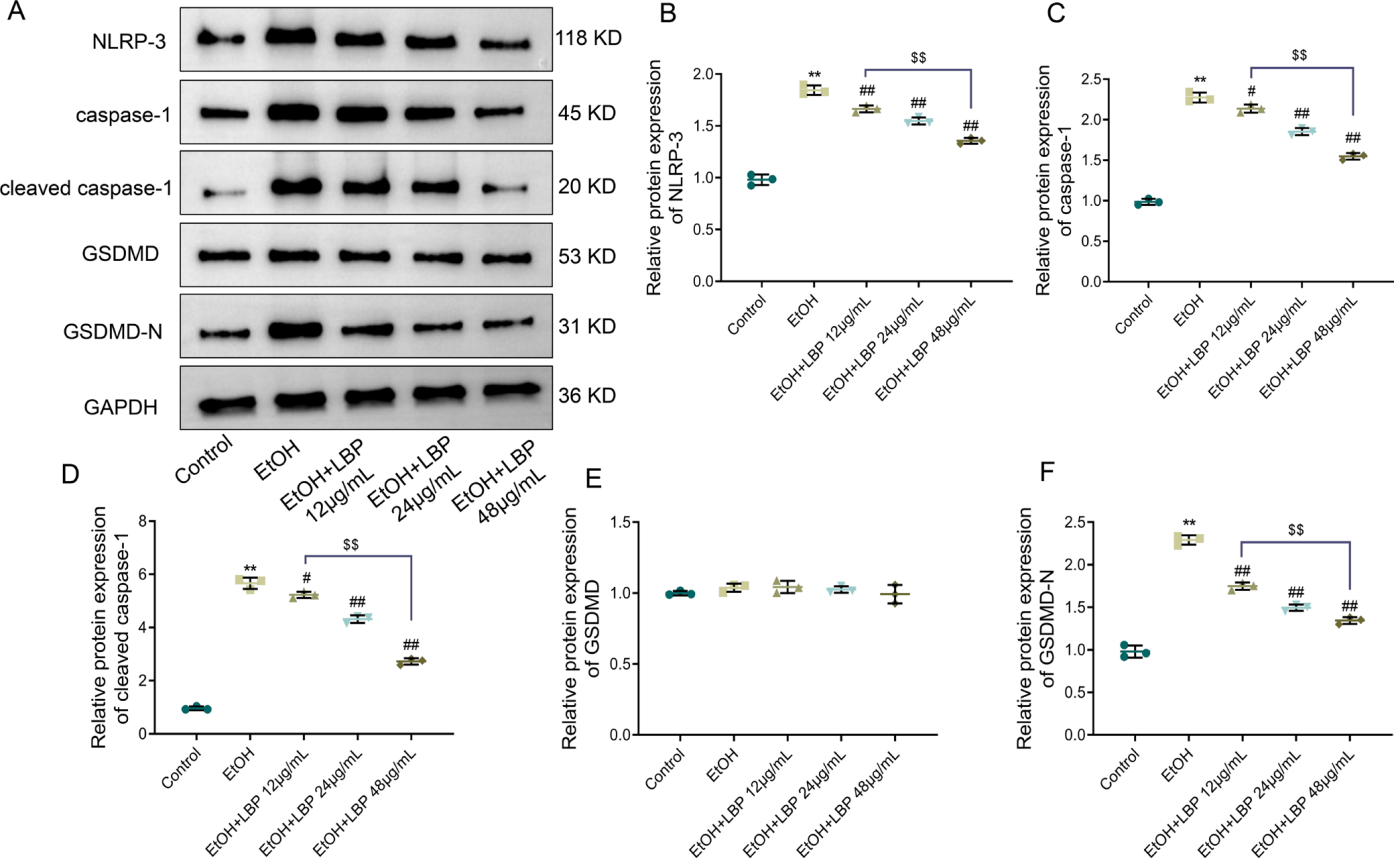
LBPs inhibit ethanol‐induced expression of pyroptosis‐related proteins in L‐02 cells. (A) Representative protein bands of NLRP‐3, cleaved caspase‐1, caspase‐1, GSDMD, and GSDMD‐N in each group. (B–F) Quantitative analysis of protein expression levels of NLRP‐3 (B), caspase‐1 (C), cleaved caspase‐1 (D), GSDMD (E), and GSDMD‐N (F). Data representation: Mean ± SD (*n* = 3). One‐way ANOVA followed by Tukey's post hoc test with Bonferroni correction was used for multiple comparisons. **p < 0.01 versus Control group; ^##^ *p* < 0.01 versus EtOH group; ^$$^ *p* < 0.01 versus EtOH+LBP 12 μg/mL group. EtOH, ethanol; GSDMD, gasdermin D; LBP, *Lycium barbarum* polysaccharide; NLRP‐3, nod‐like receptor protein 3.

We sought to determine whether LBPs could inhibit the pyroptosis‐related protein expression in L‐02 cells exposed to EtOH, given the established role of pyroptosis in EtOH‐triggered liver injury (Tien et al. 2023). The EtOH group displayed a remarkable rise in the pyroptosis‐related protein expression, including caspase‐1, cleaved caspase‐1, GSDMD‐N, and NLRP‐3, in comparison with the control group; however, the GSDMD level was raised. In contrast, LBP treatment caused a dose‐dependent and notable reduction in cleaved caspase‐1, GSDMD‐N, caspase‐1, and NLRP‐3 levels (*p* < 0.05 or *p* < 0.01). Among them, the reduction in GSDMD level was not statistically significant (Figure 3). Similarly, 48 μg/mL LBP exhibited superior regulated effects compared to lower doses (*p* < 0.01). Consequently, LBPs demonstrated the potential to inhibit EtOH‐induced pyroptosis by downregulating pyroptosis‐related protein expression in L‐02 cells, further confirming their protective role in EtOH‐triggered liver injury.

### 3.4 LBPs Stimulate EtOH‐Induced Expression of PPAR‐α in L‐02 Cells

**FIGURE 4:**
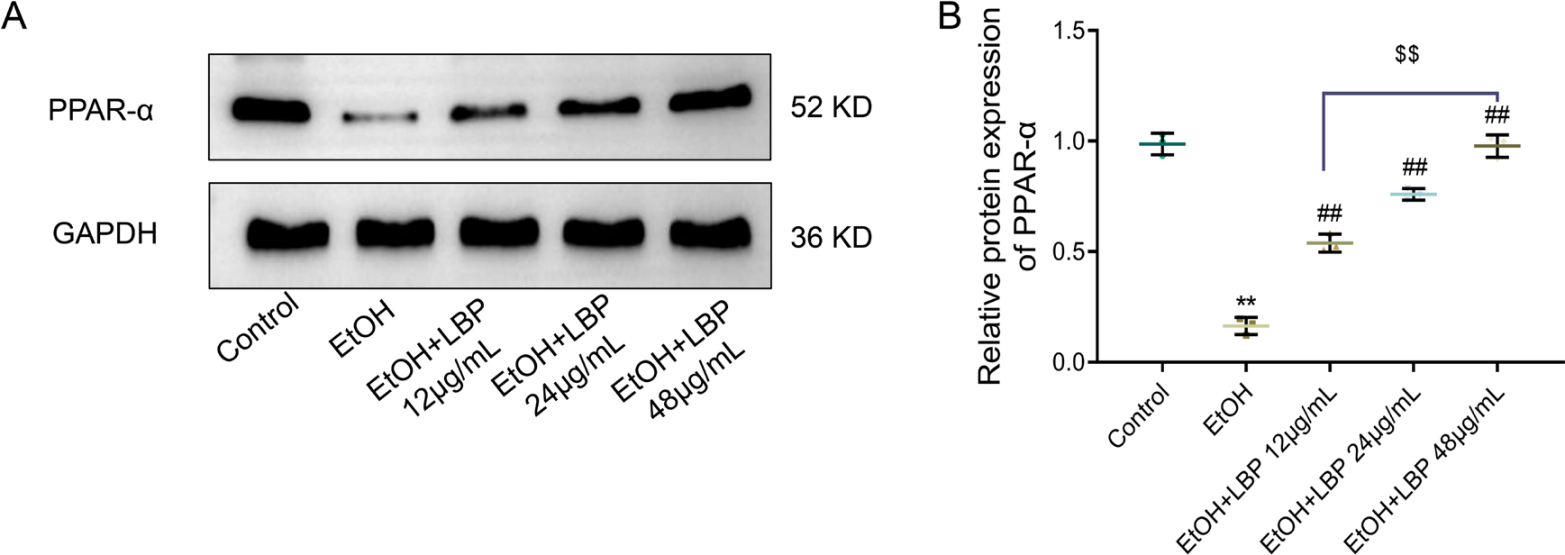
LBPs stimulate ethanol‐induced expression of PPAR‐α in L‐02 cells. (A) Representative protein band of PPAR‐α in each group. (B) Quantitative analysis of the protein levels of PPAR‐α. Data representation: Mean ± SD (*n* = 3). One‐way ANOVA followed by Tukey's post hoc test with Bonferroni correction was used for multiple comparisons. **p < 0.01 versus Control group; ^##^ *p* < 0.01 versus EtOH group; ^$$^ *p* < 0.01 versus EtOH+LBP 12 μg/mL group. PPAR‐α, peroxisome proliferator‐activated receptor‐alpha.

To investigate whether the PPAR‐α signaling can be influenced by LBPs, we examined the PPAR‐α expression in L‐02 cells with EtOH and various concentrations of LBPs. Relative to the control group, the PPAR‐α expression was considerably raised in the EtOH group (*p* < 0.001). However, in contrast to the EtOH group, LBP treatment at different concentrations (12, 24, and 48 μg/mL) resulted in a marked, dose‐dependent increase in PPAR‐α protein levels (*p* < 0.01, Figure 4). Interestingly, 48 μg/mL LBP exhibited higher protein levels as opposed to 12 μg/mL (*p* < 0.01). Therefore, LBPs effectively counteracted the EtOH‐induced reduction in PPAR‐α expression in L‐02 cells, with higher LBP concentrations correlating with greater restoration of PPAR‐α expression.

### 3.5 LBPs Attenuate EtOH‐Impelled L‐02 Cell Injury via Activating PPAR‐α

To further clarify PPAR‐α's role in LBP's protective effects, we knocked down PPAR‐α expression in L‐02 cells using siRNA and evaluated the impact on EtOH‐induced cell injury. Based on the previous experimental results, the highest concentration of LBP consistently demonstrated the most significant efficacy across all these parameters. Therefore, LBP (48 μg/mL) was chosen as the optimal dose for further validation studies to further explore the mechanisms underlying these protective effects.

**FIGURE 5:**
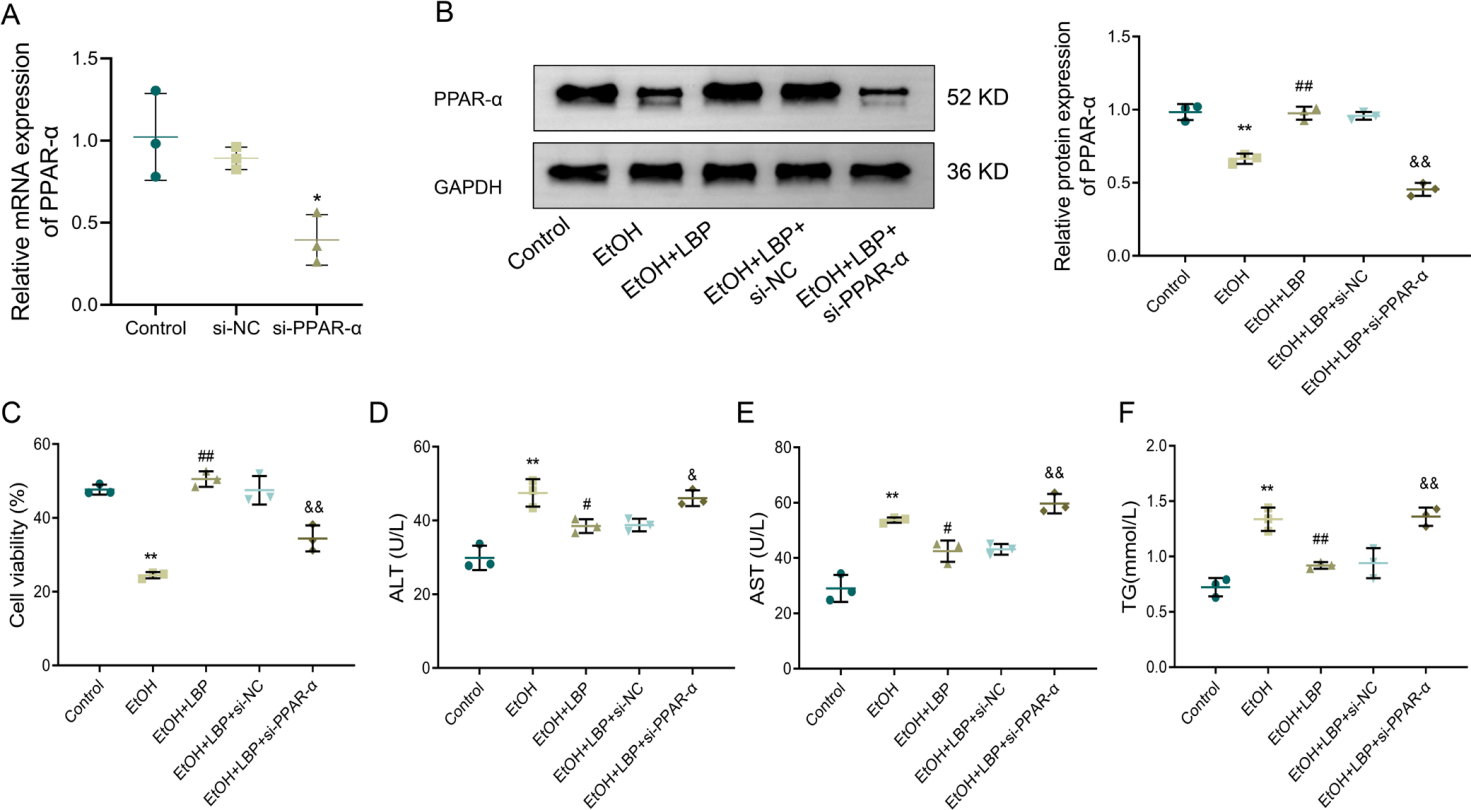
LBPs relieve ethanol‐induced L‐02 cell injury by activating PPAR‐α. (A) qRT‐PCR analysis evaluating PPAR‐α mRNA expression in each group. (B) Western blotting analysis of PPAR‐α protein expression in each group. (C) Cell viability assessed by MTT assay. (D–F) Biochemical assays measuring the levels of ALT (D), AST (E), and TG (F) in cells of each group. Data representation: Mean ± SD (*n* = 3). One‐way ANOVA followed by Tukey's post hoc test with Bonferroni correction was used for multiple comparisons. **p < 0.01 versus Control group; ^##^ *p* < 0.01 versus EtOH group; ^&&^ *p* < 0.01 versus LBP + si‐NC group; & *p *< 0.05 versus LBP + si‐NC group. ALT, alanine transaminase; AST, aspartate aminotransferase; PPAR‐α, peroxisome proliferator‐activated receptor‐alpha; TG, triglycerides.

Transfection with PPAR‐α siRNA resulted in an effective knockdown of PPAR‐α expression in L‐02 cells, as verified by qRT‐PCR (*p* < 0.05, Figure 5A). Correspondingly, as opposed to the LBP + si‐NC group, PPAR‐α protein expression was also notably dropped in the LBP + si‐PPAR‐α group (*p* < 0.001, Figure 5B). Relative to the LBP + si‐NC group, cell viability was remarkably declined in the LBP + si‐PPAR‐α group, along with a substantial increase in the levels of ALT, AST, and TG (all *p* < 0.001, Figure 5C–F). Hence, LBP's protective impacts on EtOH‐triggered L‐02 cell injury were likely mediated by activating the PPAR‐α signaling pathway.

### 3.6 LBPs Inhibit EtOH‐Induced Inflammation and Pyroptosis‐Related Protein Expression by Mediating the PPAR‐α Signaling Pathway

**FIGURE 6:**
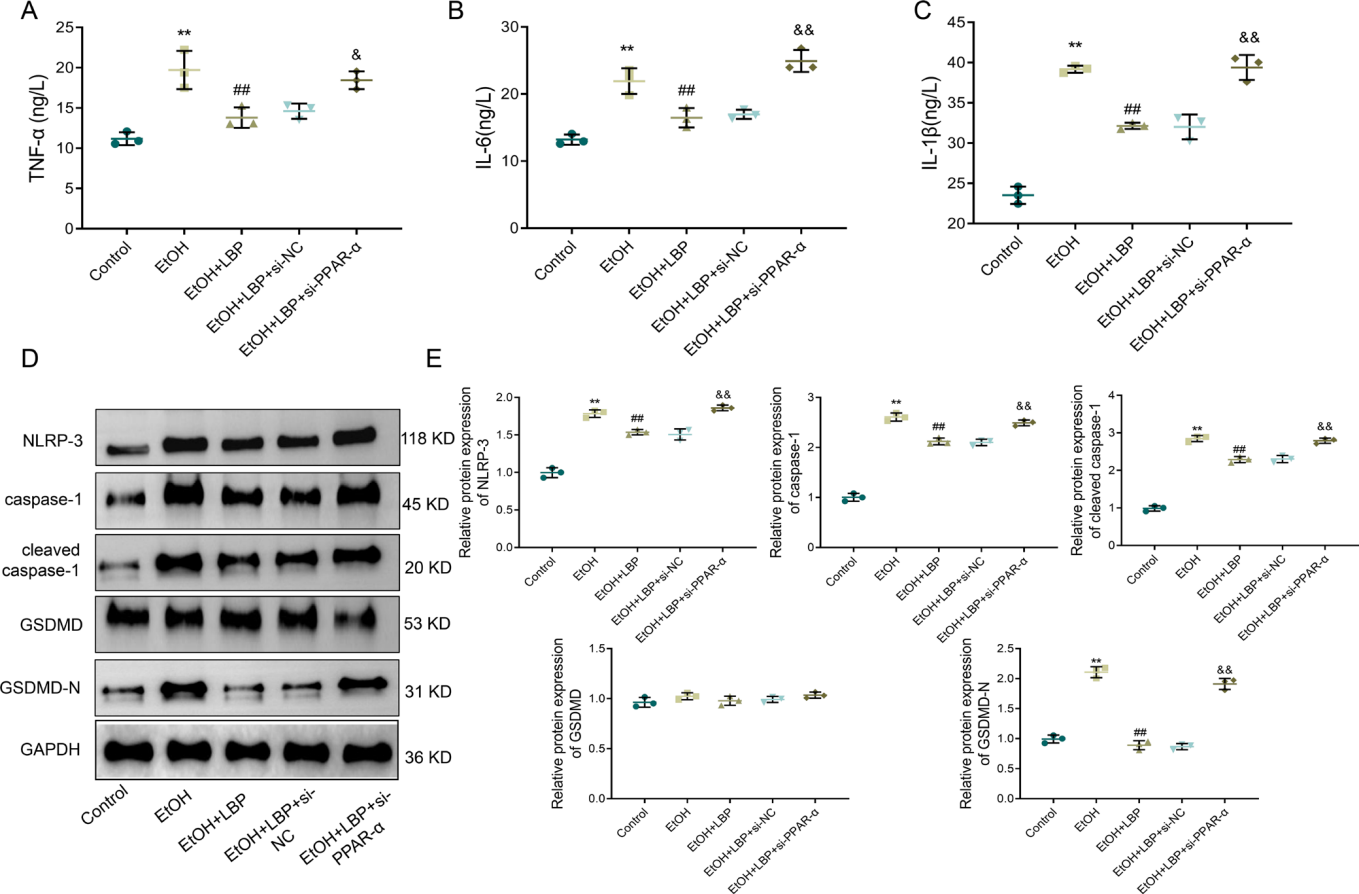
LBPs inhibit ethanol‐induced inflammation and pyroptosis‐related protein expression by mediating PPAR‐α signaling pathway. (A–C) *ELISA* results showing the levels of TNF‐α (A), IL‐6 (B), and IL‐1β (C) in cells of each group. (D) Representative protein bands of NLRP‐3, caspase‐1, cleaved caspase‐1, GSDMD, and GSDMD‐N in each group. (E) Quantitative analysis of protein levels of GSDMD, NLRP‐3, cleaved caspase‐1, caspase‐1, and GSDMD‐N. Data representation: Mean ± SD (*n* = 3). One‐way ANOVA followed by Tukey's post hoc test with Bonferroni correction was used for multiple comparisons. **p < 0.01 versus Control group; ^##^ *p* < 0.01 versus EtOH group; ^&&^ *p* < 0.01 versus LBP + si‐NC group. GSDMD, gasdermin D; IL, interleukin; NLRP‐3, nod‐like receptor protein 3; PPAR‐α, peroxisome proliferator‐activated receptor‐alpha; TNF‐α, tumor necrosis factor alpha.

We further explored whether LBP's anti‐pyroptotic and anti‐inflammatory effects on EtOH‐treated L‐02 cells are mediated by the PPAR‐α signaling. In L‐02 cells transfected with PPAR‐α siRNA, treatment with LBPs failed to reduce the EtOH‐induced upregulation of IL‐6, TNF‐α, and IL‐1β, and pyroptosis‐related proteins including cleaved caspase‐1, caspase‐1, and GSDMD‐N, relative to the LBP + si‐NC group (all *p* < 0.001, Figure 6). Similarly, the GSDMD expression (*p* > 0.05) did not significantly differ across the groups, suggesting that GSDMD might not be as critically involved in the LBP‐mediated protection against EtOH‐induced pyroptosis as the other proteins. Overall, our findings indicated that LBPs might inhibit EtOH‐induced inflammation and pyroptosis in L‐02 cells via activating the PPAR‐α signaling pathway, highlighting PPAR‐α as a critical mediator of LBP's hepatoprotective effects.

## 4 Discussion

This study was the first to demonstrate that LBPs protect against EtOH‐triggered liver injury by activating PPAR‐α and inhibiting the NLRP‐3/caspase‐1 pathway. Our results revealed a dose‐dependent effect of LBPs, with the 48 μg/mL exhibiting the most significant protection. This finding aligns with previous research on animal models, where LBPs have shown hepatoprotective impacts against liver injury caused by alcohol in rats and mice (Wang et al. 2018; Wang et al. 2020; Wei et al. 2020). By confirming these effects in the human hepatic cell model, this study extends the therapeutic relevance of LBP in ALD and suggests potential clinical applications.

The ALD pathogenesis is complex, involving oxidative stress, lipid accumulation, and inflammation, each of which contributes to liver injury (Hyun et al. 2021). In this study, LBP significantly improved cell viability and reduced the AST, TG, and ALT levels in EtOH‐treated L‐02 cells, suggesting LBP's multifaceted protective role against hepatocyte damage and lipid accumulation. The antioxidant and lipid‐lowering properties of LBPs, previously reported in the literature, likely contribute to this effect (H. Wang et al. 2020). By mitigating oxidative stress and maintaining cell membrane integrity, LBP may help reduce liver overall injury and prevent transaminase release (Fang et al. 2023; Lee et al. 2019; Yang et al. 2023).

Inflammation is vital in ALD progression, and our study demonstrates that LBP reduces the pro‐inflammatory cytokine (IL‐1β, IL‐6, and TNF‐α) levels in EtOH‐treated cells. This LBP's anti‐inflammatory effect is likely due to its ability to inhibit the NLRP‐3 inflammasome, an essential mediator of pyroptosis and inflammatory signaling in liver cells (Longo et al. 2020). Pyroptosis, distinct from apoptosis and necrosis, is an inflammatory type of cell death caused by cytokine release, caspase‐1 activation, and inflammasome assembly (Tsuchiya 2020). Specifically, caspase‐1‐mediated cleavage of GSDMD, initiated by NLRP‐3 inflammasome activation, releases its N‐terminal domain to create pores in the cell membrane. This cascade promotes cell release, rupture, and swelling of inflammatory cytokines, thereby amplifying liver inflammation and injury (Chen et al. 2023; Li et al. 2021). This research demonstrated that LBP's suppression of pyroptosis‐related proteins, including cleaved caspase‐1, GSDMD‐N, and caspase‐1, further supports its role in mitigating pyroptosis and inflammatory cell death in EtOH‐triggered liver injury (Wei et al. 2020).

Moreover, LBP likely exerted its hepatoprotective effects via regulating the PPAR‐α signaling pathway in this study. PPAR‐α, a nuclear receptor involved in inflammation, lipid metabolism, apoptosis, and pyroptosis, is downregulated in response to EtOH exposure but restored by LBP treatment in this study (Gallorini et al. 2023; Wang et al. 2021). The observed increase in PPAR‐α following LBP treatment is consistent with studies linking PPAR‐α activation to reduced liver injury through the modulation of inflammation, lipid accumulation, and pyroptosis (Chen et al. 2023; Li et al. 2021). The alignment between our outcomes and previous research highlights PPAR‐α as a potential target for ALD therapy and reinforces LBP's clinical relevance in managing liver diseases linked to lipid dysregulation and inflammation.

While our study sheds light on LBP's protective mechanisms in EtOH‐induced liver injury, there are limitations to consider. The research was conducted using the L‐02 human hepatocyte cell line, which, although informative, cannot fully replicate the complex metabolic and immunological interactions of the liver in vivo. For example, the gastrointestinal tract metabolizes LBPs extensively in vivo, generating bioactive metabolites that may differ from the intact LBPs used in this in vitro model. Consequently, these findings may not fully reflect the pharmacokinetics and bioavailability of LBP's metabolites, which could impact the therapeutic effectiveness of LBP in clinical settings. Further studies should address these limitations by including in vivo studies to validate LBP's protective mechanisms and evaluate its bioavailability and metabolite activity in a more physiologically relevant context. Additionally, examining different dosages and durations of LBP treatment could help clarify the dose–response relationship and enhance the translational potential of this research for clinical applications in ALD and related liver diseases.

In conclusion, this research demonstrates that LBPs defend against EtOH‐triggered liver injury through multiple mechanisms, including the inhibition of inflammatory responses, activation of the PPAR‐α signaling pathway, and suppression of NLRP‐3/caspase‐1‐mediated pyroptosis. These outcomes enhance the understanding of LBP's therapeutic potential in managing ALD and provide a foundation for future research into their clinical applications.

## 5 Conclusion

This study demonstrated that LBPs protected against EtOH‐induced liver injury by reducing inflammation, inhibiting NLRP‐3/caspase‐1‐mediated pyroptosis, and activating PPAR‐α signaling. These findings highlight LBPs' potential as a therapeutic agent for ALD by targeting key inflammatory and metabolic pathways. Future studies should explore their clinical applicability and long‐term efficacy in ALD management.

## Author Contributions

**Le‐bin Cai:** conceptualization (equal), data curation (equal), formal analysis (equal), visualization (equal), writing – original draft (equal), writing – review and editing (equal). **Quan Zhou:** investigation (equal), methodology (equal), project administration (equal), visualization (equal). **Na Tao:** formal analysis (equal), investigation (equal), validation (equal), visualization (equal).

## Ethics Statement

The authors have nothing to report.

## Consent

The authors have nothing to report.

## Conflicts of Interest

The authors declare no conflicts of interest.

## Data Availability

The data used to support the findings of this study are available from the corresponding author upon request.
